# Effect of long-term supplementation of low molecular weight chitosan oligosaccharide (GO2KA1) on fasting blood glucose and HbA1c in *db/db* mice model and elucidation of mechanism of action

**DOI:** 10.1186/1472-6882-14-272

**Published:** 2014-07-29

**Authors:** Jong-Gwan Kim, Sung-Hoon Jo, Kyoung-Soo Ha, Sung-Chul Kim, Young-Cheul Kim, Emmanouil Apostolidis, Young-In Kwon

**Affiliations:** Kunpoong Bio Co., Ltd., Gumnung-ri, 407–11, Hallim-eup, Jeju Special Self Governing Province, Jeju, 695-923 Korea; Department of Food and Nutrition, Hannam University, Daejeon, 305-811 Korea; Department of Nutrition, University of Massachusetts, Amherst, MA 01003 USA; Department of Chemistry and Food Science, Framingham State University, Framingham, MA 01701 USA

**Keywords:** Type 2 diabetes, Pre-diabetes, Blood glucose, Glucosidase inhibitors, Low molecular chitosan oligosacharide, GO2KA1

## Abstract

**Background:**

Type 2 diabetes is a serious problem for developed countries. Prevention of prediabetes progression to type 2 diabetes with the use of natural products appears to a cost-effective solution. Previously we showed that enzymatically digested low molecular weight chitosan-oligosaccharide with molecular weight (MW) below 1,000 Da (GO2KA1) has potential for hyperglycemia management.

**Methods:**

In this study we evaluated the effect of long-term supplementation of GO2KA1 on hyperglycemia using a *db/db* mice model. Additionally, we evaluated the effect of GO2KA1 on sucrase and glucoamylase activities and expression, using the same *db/db* mice model.

**Results:**

After 42 days we observed that GO2KA1 supplementation reduced both the blood glucose level and HbA1c in a similar manner with a known anti-diabetic drug, acarbose. When the sucrase and glucoamylase activities of GO2KA1 and control mice were evaluated using enzymatic assay, we observed that GO2KA1 significantly inhibited sucrase in all 3 parts of the intestine, while glucoamylase activity was significantly reduced only in the middle and lower part. When the sucrase-isomaltase (SI) complex expression on mRNA level was evaluated, we observed that GO2KA1 had minimal inhibitory effect on the upper part, more pronounced inhibitory effect on the middle part, while the highest inhibition was observed on the lower part. Our findings suggest that long-term GO2KA1 supplementation in *db/db* mice results to significant blood glucose and HbA1c reduction, to levels similar with those of acarbose. Furthermore, our findings confirm previous *in vitro* observations that GO2KA1 has inhibitory effect on carbohydrate hydrolysis enzymes, namely sucrase, maltase and SI complex.

**Conclusions:**

Results from this study provide a strong rationale for the use of GO2KA1 for type 2 diabetes prevention, via inhibition of carbohydrate hydrolysis enzymes. Based on the findings of this animal trial, clinical trials will be designed and pursued.

## Background

Diabetes is a group of diseases marked by high levels of blood glucose resulting from defects in insulin production, insulin action, or both [1]. Type 2 diabetes accounts for about 90% to 95% of all diagnosed cases of diabetes in adults [1]. Pre-diabetes is a condition in which individuals have blood glucose levels higher than normal but not high enough to be classified as diabetes [2]. At least 347 million people worldwide have diabetes and this figure is likely to double by 2030 [3]. In United States, in 2010, 25.8 million people (10% of American adults) had diabetes and by 2050 this figure is expected to jump to 33%, or one-third of all American adults [1]. Diabetes cost Americans $174 billion to manage in 2007 - a figure that is expected to skyrocket based on the latest estimates of the Center for Disease Control (CDC) [1].

In 2011, CDC reported that 79 million Americans (25.4% of the population) have been diagnosed as pre-diabetic [1]. The American Diabetes Association defines pre-diabetic individual as an individual with blood glucose levels higher than normal (impaired fasting glucose between 100–125 mg/dL, impaired glucose tolerance between 140–199 mg/dL, and HbA1c between 5.7-6.4%) but not high enough to be considered diabetic (impaired fasting glucose between >126 mg/dL, impaired glucose tolerance between >200 mg/dL, and HbA1c between >6.5%) [4]. α-Glucosidase inhibitors, such as acarbose and voglibose, are the only oral anti-diabetes agent approved for the treatment of pre-diabetes [5]. Briefly, lower doses of acarbose have shown to have beneficial effect towards pre-diabetes management by delaying the absorption of carbohydrates from the gut [6]. Taking into consideration that pre-diabetes is not a disease; it makes sense to identify natural components capable of reducing glucose absorption in the small intestinal environment, via inhibition of carbohydrate hydrolysis enzymes.

Hyperglycemia results from poor postprandial insulin action after dietary carbohydrate catabolism [7, 8]. Digestion of dietary carbohydrates in the distal small intestine begins with hydrolysis, which is carried out by a group of hydrolytic enzymes that includes pancreatic α-amylase and intestinal α-glucosidases [9]. Inhibition of α-glucosidase suppresses postprandial hyperglycemia by slowing down the catabolism of dietary carbohydrates [6, 10]. Recent studies showed that phenolic phytochemicals from botanical sources are natural inhibitors of α-amylase and α-glucosidase [11–14] and thus can be potentially used to manage pre-diabetes progression to type 2 diabetes.

Chitosan is a natural product commercially obtained by the deacetylation of chitin. Low molecular weight chitosan oligosaccharide results from the enzymatic digestion of chitosan and has been shown to have many health beneficial biological activities including antitumor [15, 16], immunoenhancing [17], anti-hypertension [18] and anti-diabetic [19, 20]. Specifically for type 2 diabetes management, Kondo et al. [19] showed that low molecular weight chitosan oligosaccharide can prevent the progression of diabetes in streptozotocin-induced diabetic mice. Additionally, Kim et al. [20] clinically demonstrated the blood glucose lowering effect of low molecular weight chitosan oligosaccharide in healthy human subjects. However, these studies did not try to evaluate the suggested mechanism of action. Recently, the effect of degree of chitosan hydrolysis on type 2 diabetes prevention via inhibition of carbohydrate hydrolysis enzymes was evaluated [21]. Our previous report showed that GO2KA1 (<1000 Da) had better effect for type 2 diabetes prevention in Sprague–Dawley (SD) rats, in terms of blood glucose reductions of after 1 h, resulting to a 14%, compared to the 4% reduction resulting from GO2KA3 (MW > 10,000 Da) administration [21]. The findings of this research suggest that chitosan oligosaccharide with MW < 1,000 Da has better effect towards postprandial glucose management in both animal and *in vitro* models [21].

In this study we investigate the effect of long-term supplementation and suggested mechanism of action (via inhibition of carbohydrate hydrolysis enzymes) of enzymatically digested chitosan oligosaccharide with MW < 1,000 Da (GO2KA1). Briefly, the effect of long-term supplementation of GO2KA1 in db/db mice model on glucose, HbA1c, total cholesterol and triglyceride contents was evaluated. To confirm the mechanism of action via carbohydrate hydrolyzing enzymes inhibition, after the completion of the animal trial the small intestines of the tested animal were recovered and the sucrase, glucoamylase and sucrase-isomaltase complex activities were determined.

## Methods

### Materials

Chitosan oligosaccharides classified by molecular weight (GO2KA1; MW < 1000 Da) were purchased from Kunpoong Bio Co. Ltd. (Seoul, Korea). Corn starch, casein, vitamin mix, mineral mix, calcium phosphate and sodium chloride were purchased from Raon Bio (Yonginsi, Korea). Total cholesterol and total glyceride kits were purchased from Stanbio laboratory (Boerne, USA). Blood glucose tester was purchased from Caresens (I-SENS. Inc., Anyang, Korea) and HbA1c analyzer was purchase from Infopia Inc. (Anyang, Korea).Unless noted, all chemicals were purchased from Sigma-Aldrich Co. (St. Louis, MO, USA).

### Animal and study design

In this study, ten C57BL/KsJ-*db/db*(*db/db*) mice were used under each condition. The animals were housed in individual cages in a room with a 12 h light/dark cycle (lights on from 06:00 h) with 50 ± 7% relative humidity. All rats were adapted to a meal-feeding schedule of free access to Pico 5053 diet (Oriental Bio. Co., Seongnam, Korea) with or without samples for 7 weeks. The experimental protocols were approved by the Institutional Animal Care and Use Committee (IACUC) of the Hannam University (Approval number: HNU2012-0003). The rats had free access to tap water throughout the experiment. The rats were anesthetized with pentobarbital and killed, and blood was collected. The small intestine was cut transversely into three segments (upper, middle, and lower part) of roughly equal length. Each segment was flushed with ice-cold phosphate buffered saline, frozen in liquid nitrogen, and stored at -70°C for measurement of enzyme activities.

### Blood analysis

The blood glucose level was measured with a glucose analyzer (caresensII, I-SENS Inc., Anyang, Korea) using the glucose oxidase method, and the plasma total cholesterol and total glyceride concentration was measured using a kit (Stanbio lab., Boerne, USA). Furthermore the concentration of HbA1c was measured using Nycocard reader (Anyang, Korea).

### Preparation of crude enzyme extracts

The small intestine was cut transversely into three segments (upper, middle, and lower part) of roughly equal length. Each section of intestine was homogenized in 10 mL of 100 mM potassium phosphate buffer, pH 6.8, with a homogenizer (Ultra-Turrax T25, Janke & Kunkel Co., Staufen, Germany). After centrifugation at 3000 × g for 10 min, the supernatant obtained was used as crude enzyme solution.

### Sucrase and glucoamylase activity assay

Rat-intestinal crude enzyme (1.0 g) was suspended in 3 mL of 0.9% saline, and the suspension was sonicated twelve times for 30 s with a one min time interval at 4°C. After centrifugation (10,000 g, 30 min, 4°C), the resulting supernatant was used for the assay. Sucrase and Glucoamylase activities were assayed by modifying a method developed by Dahlqvist [22]. The activity was determined by incubating a solution of crude enzyme (50 μL), 0.1 M phosphate buffer (pH 7.0, 100 μL) containing 0.4 mg/mL sucrose or soluble starch at 37°C for 30 min. The reaction mixture was heated in a boiling water bath to stop the reaction for 10 min, and then the amount of liberated glucose was measured by the glucose oxidase method.

### Western blotting analysis

The small intestine of db/db mice was dissected and lysed in a radio immuno precipitation assay (RIPA) buffer (50 mM Tris–HCl (pH 8.0), 1% NP-40, 0.5% sodium deoxycholate, 150 mM NaCl, 1 mM PMSF) that contained a phosphatase inhibitor cocktail. The lysed cells were then subjected to electrophoresis using sodium dodecylsulfate–polyacrylamide gel electrophoresis (SDS–PAGE) and transferred to nitrocellulose membranes. The membranes were reacted with primary antibodies for 3 h and then incubated with the appropriate goat peroxide-conjugated secondary antibodies for 1 h at room temperature. The proteins on the membranes were detected with a chemiluminescent detection kit (Intron Biotechnology) and visualized using the LAS4000 chemiluminescent image analyzer (Fuji, Tokyo, Japan).

### Statistical analysis

Statistical analyses were carried out using the statistical package SPSS 10 (Statistical Package for Social Science, SPSS Inc., Chicago, IL, USA) program and significance of each group was verified with the analysis of One-way analysis of variance (ANOVA) followed by the Duncan’s multiple range test of *p* < 0.05 and the Student’s *t*-test for comparison of means.

## Results

### *db/db*mice trial

**Figure 1:**
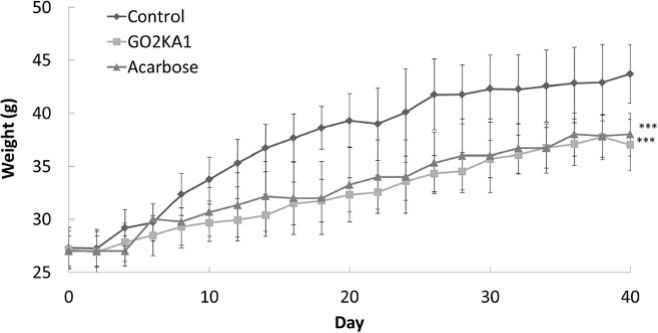
**Changes in body weight gains after administration of GO2KA1 Male** ***db/db*** **mice were free access to a high carbohydrate-diet with GO2KA1 (4%), acarbose (0.04%) and vehicle for 6 weeks.** Each point represents mean ± SD. (n = 10). *p < 0.05, **p < 0.01, and ***p < 0.001 compared to different samples at the same concentration by unpaired Student’s *t*-test.

**Figure 2:**
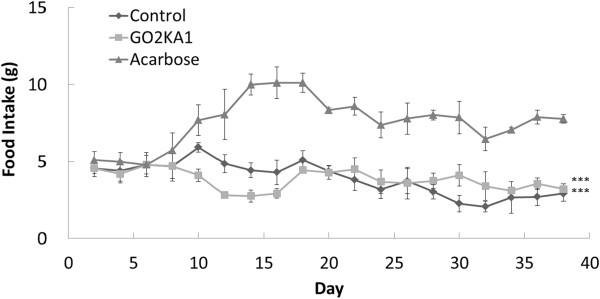
**Changes in Food Intake gains after administration of GO2KA1 Male** ***db/db*** **mice were free access to a high carbohydrate-diet with GO2KA1 (4%), acarbose (0.04%) or vehicle for 6 weeks.** Each point represents mean ± SD. (n = 10). *p < 0.05, **p < 0.01, and ***p < 0.001 compared to different samples at the same concentration by unpaired Student’s *t*-test.

The effect of GO2KA1 administration was evaluated in *db/db* mice model for 42 days and compared to the effect of acarbose, as described in the materials and methods (Table 1). After 42 days we observed that the body weight of GO2KA1 treated group was similar to acarbose treatment and significantly lower compared to control (Figure 1). Clear differentiation between control and treatments (GO2KA1 and acarbose) can be identified after 10 days of administration (Figure 1). At the same time point (42 days) the effect of GO2KA1 on fasting blood glucose, HbA1c, total cholesterol, triglyceride content and cecum weight were also evaluated (Table 2). We observed that fasting glucose levels were significantly reduced with GO2KA1 treatment to levels similar to acarbose (Table 2). More specifically fasting blood glucose levels with control were around 496 (mg/dL), while GO2KA1 and acarbose were 162.93 (mg/dL) and 150.80 (mg/dL), respectively (Table 2). Similarly, the control group had HbA1c levels around 12.58%, while GO2KA1 and acarbose resulted in significantly lower and similar levels (5.80 and 5.10%, respectively) (Table 2). Although triglyceride levels were at the same levels among all the treatments (Table 2), total cholesterol was significantly reduced only with GO2KA1 supplementation (130.65 mg/dL) while control and acarbose resulted to similar total cholesterol levels (203.30 and 180.70 mg/dL, respectively) (Table 2). Finally the cecum weight was determined at the end of the experiment and we observed that acarbose treatment group had the largest cecum (1.6 g), followed by GO2KA1 treatment (0.63 g) while control had the smallest cecum (0.36 g) (Table 2). When the food intake was evaluated in all treatments and control, we observed that the GO2KA1 and control subjects had similar levels of food consumption, while food intake dramatically increased within the acarbose group (Figure 2).

**Table 1:** **Composition of Diets (g/kg)**

High carbohydrate diets	Control (Non GO2KA1)	GO2KA1	Acarbose
Corn Starch	661	621	660.6
Casein	226	226	226
Soybean Oil	60	60	60
Vitamin Mix^1^	31	31	31
Mineral Mix^2^	9	9	9
Calcium Phospahte	10	10	10
Sodium chloride	3	3	3
Sample (GO2KA1)	0	40	0.4

^1^AIN-93VX vitamin mix (Oriental Yeast Co., Japan).

^2^AIN-93G mineral mix (Oriental Yeast Co., Japan).

**Table 2:** **Effect of GO2KA1 and acarbose treatment on various parameters in** ***db/db*** **mice**

	***db/db***mouse		
	Control	GO2KA1	Acarbose
In fasting mice			
Glucose	496.00 ± 50.02	162.93 ± 36.93***	150.80 ± 77.0***
HbA1c	12.58 ± 1.11	5.80 ± 0.80***	5.10 ± 0.30***
Total Cholesterol	203.30 ± 46.85	130.65 ± 28.20***	180.70 ± 25.60
Triglyceride	137.91 ± 12.60	136.33 ± 6.33	123.90 ± 16.40
Cecum	0.36 ± 0.05	0.63 ± 0.18***	1.60 ± 0.40***

Each point represents mean **±** SD. (n = 10). *p < 0.05, **p < 0.01, and ***p < 0.001 compared to different samples at the same concentration by unpaired Student’s *t*-test.

### Mice intestinal sucrase and glucoamylase activities

**Figure 3:**
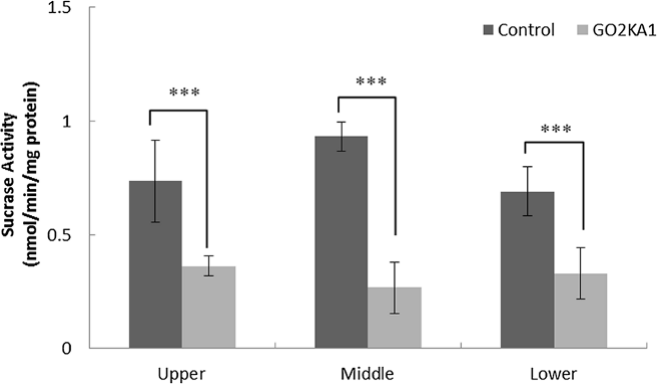
**Effects of GO2KA1 administration on sucrase activities (nmol/min/mg protein) in different parts of intestine.** The results represent the mean ± S.D. of values obtained from three measurements. Each point represents mean ± SD. (n = 10). *p < 0.05, **p < 0.01, and ***p < 0.001 compared to different samples at the same concentration by unpaired Student’s *t*-test.

**Figure 4:**
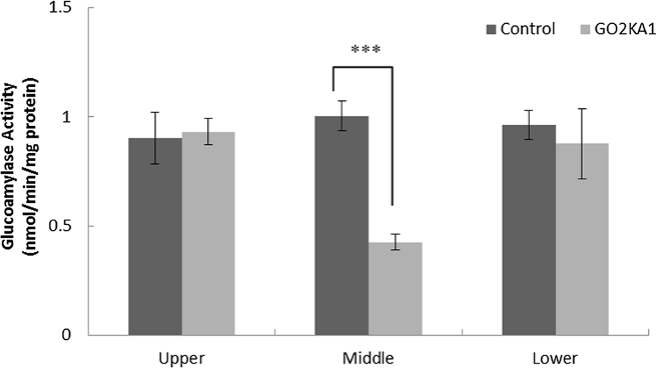
**Effects of GO2KA1 administration on glucoamylase activities (nmol/min/mg protein) in different parts of intestine.** The results represent the mean ± S.D. of values obtained from three measurements. Each point represents mean ± SD. (n = 10). *p < 0.05, **p < 0.01, and ***p < 0.001 compared to different samples at the same concentration by unpaired Student’s *t*-test.

To confirm the extent of carbohydrate hydrolyzing enzyme inhibition with GO2KA1 treatment samples were prepared and analysed as outlined in the materials and methods and analysed for their sucrase and glucoamylase activity at the three different small intestinal regions (Upper part – duodenum, Middle part – jejunum and Lower part – ileum). Our observations suggest that sucrase activity is significantly reduced in throughout the small intestine (Figure 3). However, glucoamylase activity is reduced only in the jejunum, while the determined activities in the duodenum and ileum are similar (Figure 4).

### Mouse intestinal sucrase-isomaltase (SI) complex mRNA expression

**Figure 5:**
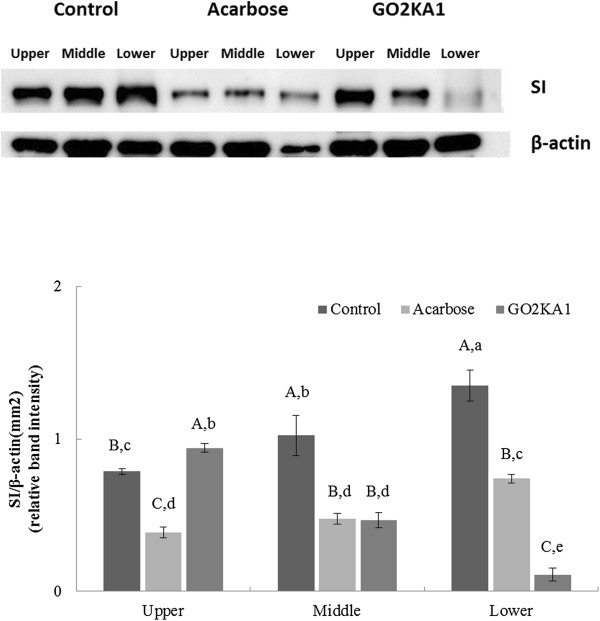
**Effect of GO2KA1 and acarbose on the SI complex (Sucrase-isomaltase complex) mRNA expression at the 3 different parts of the intestine.** The results represent the means ± S.D. of values obtained from three measurements. Different corresponding letters indicate significant differences at *p* < 0.05 by Duncan’s test. First letter is among different samples and second one is among different concentrations within same samples.

The effect of acarbose and GO2KA1 supplementation on mRNA expression of mouse intestinal sucrase-isomaltase (SI) complex was evaluated using Western blot analysis. We observed that acarbose significantly reduced SI expression in all three intestinal parts (Figure 5). However, in GO2KA1-fed group, SI expression was affected in a different manner. We observed that in the upper part SI expression was slightly up-regulated (Figure 5), when compared to control. In the middle part, the expression was significantly reduced, compared to control and it was in the same levels with the acarbose treated group (Figure 5). In the lower part, the expression was significantly reduced when compared to both control and acarbose treated group (Figure 5).

## Discussion

According to the Center for Disease Control (CDC), 79 million Americans (25.4% of the population) have been diagnosed as pre-diabetic [1]. Prevention of the progression of pre-diabetes to type 2 diabetes using natural products is an appealing strategy to control the incidence of diet-linked hyperglycaemia. Recent findings suggest that enzymatically-digested chitosan oligosaccharide with MW < 1,000 Da (GO2KA1) has better effect towards postprandial glucose management in both animal and *in vitro* models [21]. In this study we evaluated the effect of long-term administration of GO2KA1 and the possible mechanism of action using *db/db* mice model.

The effect of GO2KA1 administration was evaluated in *db/db* mice model for 42 days and compared to the effect of acarbose, as described in the materials and methods. We observed that the body weight, fasting glucose levels and HbA1c levels of GO2KA1 treated group was similar to acarbose treatment and significantly lower compared to control (Figure 1, Table 2). The above findings suggest that GO2KA1 is preventing the progression of obesity and diabetes due to carbohydrate-rich diet in *db/db* mice, in similar manner to the known α-glucosidase inhibitor, acarbose. Our observations suggest that GO2KA1 behaves in a similar manner to the known anti-diabetic drug acarbose in *db/db* mice model (Figure 1, Table 2), without having the side-effect of excessive α-glucosidase inhibition observed with the acarbose treatment that results to significantly increased cecum weight (Table 2) and increased food intake (Figure 1). However, based strictly on cecum observations, we can suspect that GO2KA1 supplementation results to moderate inhibition of carbohydrate hydrolyzing enzymes that results to slight increase of cecum weight when compared to the control (Table 2).

Furthermore, we evaluated the effect of GO2KA1 supplementation on *db/db* mice intestinal sucrase and glucoamylase activities (Figures 3 and 4). Our observations suggest that GO2KA1 administration resulted in reduced sucrase and glucoamylase activities (Figures 3 and 4). It is well-documented that acarbose binds with high affinity and specificity to α-glucosidases found in the brush border of the small intestine [5, 9, 23]. However, when acarbose is used at lower doses for prevention of pre-diabetes progression to type 2 diabetes, the resulting effect is milder inhibition of glucosidases throughout the small intestine to eventually retard glucose uptake [5]. Our observations indicate that GO2KA1 has strong inhibitory activity against sucrase and mild effect against glucoamylase (Figures 3 and 4).

When the effect of both acarbose and GO2KA1 were evaluated on mouse intestinal SI complex, on mRNA level, we observed that acarbose resulted in reduced expression in all three parts of the intestine, while GO2KA1 administration resulted to reduced expression only in the middle and lower parts (Figure 5). These results suggest that GO2KA1 has milder effect on SI activity, when compared to acarbose.

It is important to note that GO2KA1 administration resulted to almost 3 times lower cecum weight (Table 2). The major side effect of acarbose administration is flatulence and diarrhoea resulting from the excessive inhibition of starch breakdown. This results to increased cecum weight. This inhibition of pancreatic α-amylase by acarbose may induce major adverse effects such as abdominal distention, flatulence, meteorism, and diarrhea a consequence of undigested carbohydrates entering the colon where they are used as nutrients for bacterial growth [24, 25]. The differences in cecum weight and volume among the control, acarbose, and GO2KA1 groups are shown in Table 2. Acarbose administration resulted in a 3-fold increase in the weight and volume of the cecum compared with the control and GO2KA1, which is consistent with a previous study [24, 25]. Our findings indicate that GO2KA1 administration has milder (if any) side-effects when compared to acarbose.

Our observations suggest that GO2KA1 supplementation in *db/db* mice along with high starch diet results to fasting blood glucose level, HbA1c and total weight reductions to a similar level as acarbose (Figure 1, Table 2). The suggested mechanism of action is via inhibition on small intestinal α-glucosidases (Figures 4 and 5) and GO2KA1 and acarbose administration has similar effect on fasting blood glucose levels, HbA1c and body weight (Figure 1, Table 2).

## Conclusions

In this manuscript we report that GO2KA1 can effectively manage the fasting blood glucose and HbA1c levels in *db/db* mice, in a similar manner to acarbose. Here we show in an animal model that the mechanism involves inhibition of carbohydrate hydrolysis enzymes. Our findings provide evidence for the potential application of GO2KA1 for the management of type 2 diabetes, that need to be further confirmed in a clinical level.
